# Characterizing idiopathic pulmonary fibrosis patients using US Medicare-advantage health plan claims data

**DOI:** 10.1186/s12890-018-0759-5

**Published:** 2019-01-10

**Authors:** Kathleen Mortimer, Nadine Hartmann, Christine Chan, Heather Norman, Laura Wallace, Cheryl Enger

**Affiliations:** 1 Optum Epidemiology, Boston, MA USA; 2 Boehringer Ingelheim International GmbH, Ingelheim, Germany; 3 Boehringer Ingelheim Pharmaceuticals International, Ridgefield, CT USA; 4 Optum Epidemiology, 315 E. Eisenhower Parkway, Suite 305, Ann Arbor, MI 48108 USA

**Keywords:** Idiopathic pulmonary fibrosis, Claims data, Elderly, Medicare

## Abstract

**Background:**

Idiopathic pulmonary fibrosis (IPF) is a rare life-threating interstitial lung disease (ILD). This study characterizes demographics, health care utilization, and comorbidities among elderly IPF patients and estimates prevalence and incidence rates for selected outcomes.

**Methods:**

Cohort study using a large US health insurance database (Optum’s Medicare Advantage plan). Inclusion criteria: ≥ 1 diagnosis code for IPF (2008 - 2014), age ≥65 years, no diagnosis of IPF or other ILD in prior 12 months. Demographics, health care utilization, comorbidities and incidence rates for various outcomes were estimated. Follow-up continued until the earliest of: health plan disenrollment, death, a claim for another known cause of ILD, or end of the study period.

**Results:**

4,716 patients were eligible; 53.4% had IPF diagnostic testing. Median age was 77.5 years, 50.3% were male, median follow-up time was 0.8 years. Incidence rates ranged from 1.0/1,000 person-years (lung transplantation) to 374.3/1,000 person-years (arterial hypertension). Baseline characteristics and incidence rates were similar for cohorts of patients with and without IPF diagnostic testing.

**Conclusions:**

Elderly IPF patients experience a variety of comorbidities before and after IPF diagnosis. Therapies for IPF and for the associated comorbidities may reduce morbidity and associated health care utilization of these patients.

**Electronic supplementary material:**

The online version of this article (10.1186/s12890-018-0759-5) contains supplementary material, which is available to authorized users.

## Background

Idiopathic pulmonary fibrosis (IPF) is a rare interstitial lung disease (ILD) which is progressive and life-threatening. It generally occurs in patients over the age of 50 years. The diagnosis is challenging as IPF shares symptoms with many other kinds of lung disease, but improvements in diagnosis have been made [1, 2]. Diagnostic criteria include the presence of a specific radiologic pattern of usual interstitial pneumonia (UIP) on HRCT, or specific combinations of radiologic and histopathologic patterns in patients who have undergone a surgical lung biopsy [1, 3]. Nintedanib and pirfenidone were both approved for use in the United States (US) by the Food and Drug Administration (FDA) in October 2014. These were the first pharmacological treatments shown to be efficacious in patients with IPF, leading to slower disease progression [4, 5]. The objective of this study was to characterize the IPF population aged 65 years and above prior to the marketing of pharmaceutical treatments for IPF to gain a better understanding of the characteristics and disease outcomes.

## Methods

### Data Source

This non-interventional cohort study was based on a proprietary research database containing eligibility, pharmacy claims, and medical claims data from a Medicare Advantage and Part D plan (MAPD) managed by Optum. Medical and pharmacy claims data are available starting in 2006 for approximately 4.2 million members. Medicare Advantage plans are offered by private companies, such as the health insurer associated with Optum, and approved by Medicare.

Medical and pharmacy information, including inpatient and outpatient procedures and diagnoses, is available for Medicare enrollees with medical and pharmacy coverage. Pharmacy claims contain sufficient information to trace patients’ pharmacy expenditures through the multiple phases of the Part D plans, although information on medications administered during inpatient stays is not captured. All data access conforms to applicable Health Insurance Portability and Accountability Act policies.

To identify possible out-of-hospital deaths, claims data were linked to the Social Security Administration (SSA) Death Master File (DMF), a compilation of mortality information derived from the US SSA payment records. The DMF currently contains over 94 million records. Information on cause of death is not included.

### Cohort Identification

Patients with at least one medical claim with a diagnosis code of IPF and aged 65 years or older between 01 January 2008 and 30 September 2014 were identified. The ICD-9 code for IPF was 516.3 prior to October 2011, and changed to 516.31 in October 2011. Patients were required to have complete medical coverage and pharmacy benefits and at least 12 months of continuous health plan enrollment prior to the IPF claim date (baseline period).

The cohort entry date (index date) was set as the date of the first medical claim with a diagnosis of IPF within the study period. Only incident cases, defined as IPF patients without IPF claims in the baseline period were eligible for this study. Patients with other known causes of ILD such as connective tissue disease, hypersensitivity pneumonitis and others during baseline were excluded (Additional file 1, [3]). Patients with claims for IPF diagnostic testing (high-resolution computed tomography (HRCT) of the thorax or surgical lung biopsy (SLB) during baseline) were identified for a subgroup analysis (referred to as the IPF diagnostic testing subgroup). This was a stricter case definition done to identify a subgroup who would more definitively be considered to have IPF, to assess how the case definition may influence the results. The number of patients with IPF diagnostic testing in the first 30 days after diagnosis was also evaluated.

Since IPF shares symptoms with many other lung diseases, it is possible for a diagnosis of IPF to be made, then later modified to an alternate ILD diagnosis. These patients are included in the primary analysis so as not to use future events to classify patients at baseline). For exploratory purposes, however, a sensitivity analysis excluding patients who had at least one claim during follow-up for one of the ILD conditions (listed in Additional file 1) was done to estimate the impact of these potentially misdiagnosed members of the cohort on the baseline characteristic distributions and outcomes.

### Baseline characteristics

Baseline characteristics include age at cohort entry, sex, geographic area, race, length of health plan membership prior to cohort entry, cohort entry year, medication dispensings of: oral corticosteroids, N-acetyl cysteine (NAC), azathioprine and cyclophosphamide, open lung biopsies, oxygen therapy, medication for gastroesophageal reflux disease (GERD) therapy (*e.g.,* H2 receptor blockers, proton pump inhibitors), anticoagulation (*e.g.,* vitamin K antagonists, heparin, novel oral anticoagulants and antiplatelet therapy), dispensings of drugs that may induce pulmonary fibrosis (amiodarone, bleomycin, nitrofurantoin, methotrexate, gold salts), Epstein-Barr virus (EBV), hepatitis C, and broncho-alveolar lavage. Conditions defined in the list of primary and secondary outcomes below were also included in the list of baseline conditions, as were measures of health care utilization (HCRU) such as number of inpatient and outpatient visits and associated costs.

### Outcomes

Primary outcomes include acute respiratory worsening of unknown cause (ARWUC) [6], pulmonary hypertension (PH) [7], pulmonary arterial hypertension (PAH) [7], lung transplantation, lung cancer [8, 9], acute myocardial infarction (AMI), and all-cause mortality. Secondary outcomes include gastrointestinal (GI) perforation [10], chronic renal failure/insufficiency [11, 12], hemorrhagic diathesis or coagulopathy, venous thrombosis [13], pulmonary embolism [7], stroke, cardiac arrhythmia [13], congestive heart failure [14], ischemic heart disease [15, 16], arterial hypertension [17, 18], neutropenia [19], pneumonia [20], sepsis [21], chronic obstructive pulmonary disease (COPD) [22], GERD [23, 24], type 2 diabetes mellitus [25], obstructive sleep apnea, bronchitis, upper respiratory tract infection, pulmonary rehabilitation, acute coronary syndrome, angina pectoris, bleeding (including major upper and lower GI bleeding, hemorrhage of the rectum or anus, blood in stool, epistaxis, hemorrhoids, hemorrhoids with bleeding, intracranial hemorrhage), acute pancreatitis, hepatic failure, acute renal failure and two definitions of depression, including major depressive disorder (MDD) only, and MMD and other types of depression.

When available, algorithms that have been validated in administrative databases were used to define outcomes of interest. Otherwise, outcome definitions were determined with clinical input and searches of medical claims coding systems.

Follow-up time for each cohort member extended from the day after the index date until the earliest of disenrollment from the health plan, death, occurrence of baseline exclusion criterion during follow-up, or the end of the study period. For each outcome, patients were censored for any second occurrences of that outcome, but remained eligible for a different outcome.

### Statistical Methods

Mean, standard deviation (SD), median and interquartile ranges (IQR) were calculated for continuous variables and absolute and relative frequencies were calculated for categorical variables.

For the baseline characteristics, relative frequencies (prevalences) were calculated by dividing the number of patients in the cohort with the condition during baseline by the total number of patients in the cohort. Incidence rates (IRs) were calculated by dividing the number of patients with the outcome by the sum of all observation time-to-event or censoring for all patients within each cohort. IRs are presented per 1,000 person-years (pys) with 95% confidence intervals (CIs). They are shown only for the patients who did not have evidence of the condition during baseline. Length of follow-up (mean, SD, median, interquartile range (IQR)) was summarized by reason for censoring.

All analyses were conducted using SAS 9.4.

## Results

A total of 4,716 incident cases of IPF were identified. The median age of these patients was 77.5 years (IQR: 72.0, 82.0), 50.3% were male, and the majority (74.1%) were white. Approximately half (n=2,223, 47.1%) entered the cohort before October 2011, and 2,493 (53.9%) patients entered the cohort after the change in ICD-9 coding in October 2011. The most commonly observed pre-specified baseline medications were GERD therapies (34.9%) and oral corticosteroids (33.4%) (Table 1). Within the IPF cohort, 53.4% (n=2,518) had IPF diagnostic testing, of which 97.7% of the patients had claims for HRCT testing only, 0.3% had only surgical lung biopsy claims, and 2.0% had claims for both HRCT and surgical lung biopsy (data not shown). When the time period for possible diagnostic testing was extended beyond baseline to include the 30 days after the cohort entry date, an additional 437 patients were identified with IPF diagnostic testing (data not shown).

**Table 1:** Baseline characteristics of IPF Patients

	IPF Cohort (*N*= 4,716)		IPF Diagnostic Testing Subgroup (*N*= 2,518)	
Age (continuous, years)				
Median, IQR	77.5	72.0 - 82.0	77.0	71.0 - 81.0
Mean, SD	76.8	5.9	76.3	5.8
Length of Health Plan Membership Prior to Cohort Entry (continuous, months)				
Median, IQR	34.3	21.5 - 58.6	34.7	21.6 - 59.1
Mean, SD	47.6	37.9	48.1	38.4
	N	%	N	%
Age (years)				
65 – 74	1,683	35.7	981	39.0
75 – 84	2,633	55.8	1,366	54.2
85 +^a^	400	8.5	171	6.8
Sex				
Male	2,374	50.3	1,319	52.4
Female	2,342	49.7	1,199	47.6
Geographic Area				
Northeast	821	17.4	467	18.5
Midwest	1,665	35.3	909	36.1
South	1,776	37.7	911	36.2
West	454	9.6	231	9.2
Race				
White	3,496	74.1	1,899	75.4
African American	489	10.4	234	9.3
Hispanic/Latino	302	6.4	170	6.8
Asian	113	2.4	49	1.9
Other	316	6.7	166	6.6
Cohort Entry Period	N	%	N	%
2008 - 2009	970	20.6	525	20.8
2010 - 2011	1,401	29.7	731	29.0
2012 - 2014	2,345	49.7	1,262	50.1
Patients with at Least One				
Diagnosis, Procedure, or				
Dispensing for each of the				
Following During the 12-Month Baseline Period:				
Any Corticosteroid	1,577	33.4	952	37.8
NAC	40	0.8	24	1.0
Azathioprine	27	0.6	15	0.6
Cyclophosphamide	13	0.3	11	0.4
Open Lung Biopsies	22	0.5	22	0.9
Oxygen Therapy	1,093	23.2	654	26.0
GERD Therapy	1,645	34.9	912	36.2
Anticoagulation/Antiplatelet Therapy	1,346	28.5	781	31.0
Amiodarone	172	3.6	107	4.2
Bleomycin	2	0.0	2	0.1
Nitrofurantoin	207	4.4	95	3.8
Methotrexate	15	0.3	8	0.3
Gold Salts	0	0.0	0	0.0
Epstein-Barr Virus	9	0.2	7	0.3
Hepatitis C	105	2.2	73	2.9
Bronchial Lavage	150	3.2	139	5.5

Optum Research Database - Medicare Advantage Population. Cohort Entry: 01 January 2008 - 30 September 2014

Abbreviations: *IPF* idiopathic pulmonary fibrosis, *NAC* N-acetyl cysteine, *IQR* interquartile range, *SD* standard deviation, *GERD* gastroesophageal reflux disease

^a^For privacy reasons, the earliest year of birth in the MAPD is coded as 1927, so no patients in the cohort entry period prior to 2011 can be coded as age >= 85. Counts for patients who entered the cohort before October 2011 and whose actual age is >= 85 are collapsed into the 75-84 year grouping in this report

The baseline characteristics of the IPF cohort and subgroup with IPF diagnostic testing during baseline were similar, overall and within stratum of cohort entry time based on changes in ICD-9 coding (*i.e.,* cohort entry before October 2011 vs during or after October 2011). Due to the similarity of characteristics, all results are reported without stratification by cohort entry time.

Baseline HCRU metrics are summarized in Table 2. Half of the IPF cohort (50.8%) was hospitalized and 82.9% had dispensings for at least 3 unique medications. The median number of physician visits was 12.0 (IQR: 8.0-19.0). The median total cost was $11,865 (IQR: 2,465-25,113). Nearly half of the costs were from facility charges, which is consistent with the observed proportion of patients with inpatient hospitalizations.

**Table 2:** Health care utilization among IPF patients during the 12-month baseline period

	IPF Cohort (*N*= 4,716)			IPF Diagnostic Testing Subgroup (*N*= 2,518)		
	N	%		N	%	
No Medication within 12 Months of Cohort Entry	570	12.1		298	11.8	
One Medication within 12 Months of Cohort Entry	136	2.9		59	2.3	
Two Medications within 12 Months of Cohort Entry	100	2.1		46	1.8	
Three or More Medications within 12 Months of Cohort Entry	3,910	82.9		2,115	84.0	
Any Hospitalization within 12 Months of Cohort Entry (yes/no)	2,396	50.8		1,401	55.6	
Critical Care Evaluation and Management	722	15.3		447	17.8	
	Mean (SD)	Median	IQR	Mean (SD)	Median	IQR
Number of Physician Visits^a^	14.8 (10.8)	12.0	8.0 - 19.0	16.2 (11.6)	14.0	9.0 - 21.0
Number of Emergency Department Visits^a^	1.4 (2.2)	1.0	0.0 - 2.0	1.5 (2.4)	1.0	0.0 - 2.0
Number of Inpatient Stays	0.9 (1.2)	1.0	0.0 - 1.0	1.0 (1.3)	1.0	0.0 - 1.0
Number of Inpatient Days	7.2 (18.0)	1.0	0.0 - 6.0	8.4 (19.3)	1.0	0.0 - 8.0
Number of 3-Digit Diagnosis Codes	31.6 (14.7)	29.0	21.0 - 40.0	34.4 (14.9)	32.0	23.0 - 43.0
Number of Surgical Procedures^a^	7.6 (7.2)	6.0	3.0 - 10.0	8.4 (7.9)	6.0	3.0 - 11.0
Number of Anesthesia Procedures^a^	0.5 (0.9)	0.0	0.0 - 1.0	0.5 (0.9)	0.0	0.0 - 1.0
Number of Unique Drugs Dispensed	10.3 (7.4)	10.0	5.0 - 15.0	10.8 (7.7)	10.0	5.0 - 16.0
Medical Costs ($)	6,033.9 (9,319.3)	3,932.7	2,156.7 - 6,986.0	6,983.1 (9,791.5)	4,479.6	2,684.1 - 7,864.9
Facility Costs ($)	12,474.9 (22,355.4)	4,845.6	864.9 - 15,263.1	15,086.9 (26,551.3)	6,430.2	1,424.9 - 18,539.2
Pharmacy Costs ($)	2,675.3 (5,854.3)	1,350.8	326.7 - 3,186.2	2,787.4 (5,989.5)	1,463.7	355.3 - 3,238.8
Total Costs ($)	21,184.2 (28,913.5)	11,865.2	5,465.4 - 25,713.4	24,857.5 (33,600.8)	13,798.3	6,739.4 - 30,886.0

Optum Research Database – Medicare Advantage Population. Cohort Entry: 01 January 2008 - 30 September 2014

Abbreviations: *IR* incidence rate per 1,000 person-years, *IQR* interquartile range, *SD* standard deviation, *CI* confidence interval, *IPF* idiopathic pulmonary fibrosis, *GERD* gastroesophageal reflux disease, *GI* gastrointestinal, *MDD* major depressive disorder

^a^One of each type counted per day

The mean length of time from cohort entry until censoring was 1.3 years (SD 1.4, median 0.8) (Table 3). The most common reason for censoring (other than the end of the study period) was the end of health plan enrollment (27.3% and 26.3%, for IPF cohort and IPF diagnostic testing subgroup). The smallest proportion of patients in each cohort was censored because one of the baseline exclusion criteria (*e.g.,* other known causes of ILD) was observed during follow-up (17.1% and 19.1%, respectively.)

**Table 3:** Length of follow-up among IPF patients

Cohorts	Length of Follow-Up (in Years)				
	N	%	Mean (SD)	Median	Interquartile Range
IPF Cohort	4,716	100.0	1.3 (1.4)	0.8	0.2 - 1.8
Reason for Censoring:					
End of Study Period	1,537	32.6	1.8 (1.7)	1.3	0.5 - 2.7
Death	1,082	22.9	0.9 (1.1)	0.5	0.1 - 1.4
End of Health Plan Enrollment	1,289	27.3	1.2 (1.2)	0.9	0.3 - 1.8
Exclusion Criteria Observed	808	17.1	0.8 (1.0)	0.4	0.1 - 1.1
IPF Diagnostic Testing Subgroup	2,518	100.0	1.2 (1.4)	0.7	0.2 - 1.7
Reason for Censoring:					
End of Study Period	793	31.5	1.8 (1.7)	1.2	0.5 - 2.7
Death	584	23.2	0.9 (1.1)	0.4	0.1 - 1.2
End of Health Plan Enrollment	661	26.3	1.2 (1.2)	0.8	0.3 - 1.8
Exclusion Criteria Observed	480	19.1	0.7 (0.9)	0.3	0.1 - 1.0

Optum Research Database – Medicare Advantage Population. Cohort Entry: 01 January 2008 - 30 September 2014

Abbreviations: *IPF* idiopathic pulmonary fibrosis, *SD* standard deviation

The baseline prevalence of the primary and secondary conditions are presented in Table 4. With the exception of lung cancer (16.1%), the prevalence was low for the primary conditions, ranging from 0.2% for PAH and lung transplantation to 4.6% for PH in the IPF cohort. Among the secondary conditions, the prevalence ranged from ≤1% for GI perforation, neutropenia, hemorrhoids with bleeding, intracranial hemorrhage, acute pancreatitis, and hepatic failure to the highest prevalence estimate of 76.3% for arterial hypertension. The results were similar for the IPF diagnostic testing subgroup.

**Table 4:** Prevalence of comorbidities during the 12-month baseline period for the newly diagnosed IPF cohorts

	IPF Cohort (*N*= 4,716)			IPF Diagnostic Testing Subgroup (*N*= 2,518)		
	# of Patients with Condition During Baseline	Prevalence (%)	95% CI	# of Patients with Condition During Baseline	Prevalence (%)	95% CI
Primary						
Acute Respiratory Worsening of Unknown Cause	126	2.7	2.2 - 3.1	126	5.0	4.2 - 5.9
Pulmonary Hypertension	219	4.6	4.0 - 5.2	161	6.4	5.4 - 7.3
Pulmonary Arterial Hypertension	10	0.2	0.1 - 0.3	5	0.2	0.0 - 0.4
Lung Transplantation	10	0.2	0.1 - 0.3	2	0.1	0.0 - 0.2
Lung Cancer	757	16.1	15.0 - 17.1	493	19.6	18.0 - 21.1
Acute Myocardial Infarction^a^	125	2.7	2.2 - 3.1	78	3.1	2.4 - 3.8
Acute Myocardial Infarction^b^	128	2.7	2.3 - 3.2	72	2.9	2.2 - 3.5
Secondary						
GI Perforation	11	0.2	0.1 - 0.4	6	0.2	0.0 - 0.4
Chronic Renal Failure/Insufficiency	1,250	26.5	25.2 - 27.8	718	28.5	26.8 - 30.3
Hemorrhagic Diathesis or Coagulopathy	120	2.5	2.1 - 3.0	80	3.2	2.5 - 3.9
Venous Thrombosis	304	6.4	5.7 - 7.1	190	7.5	6.5 - 8.6
Pulmonary Embolism	155	3.3	2.8 - 3.8	98	3.9	3.1 - 4.6
Stroke^c^	179	3.8	3.3 - 4.3	91	3.6	2.9 - 4.3
Stroke^d^	79	1.7	1.3 - 2.0	39	1.5	1.1 - 2.0
Cardiac Arrhythmia	1,612	34.2	32.8 - 35.5	935	37.1	35.2 - 39.0
Congestive Heart Failure	1,495	31.7	30.4 - 33.0	849	33.7	31.9 - 35.6
Ischemic Heart Disease	1,906	40.4	39.0 - 41.8	1,073	42.6	40.7 - 44.5
Arterial Hypertension	3,599	76.3	75.1 - 77.5	1,941	77.1	75.4 - 78.7
Neutropenia	40	0.8	0.6 - 1.1	32	1.3	0.8 - 1.7
Pneumonia	436	9.2	8.4 - 10.1	286	11.4	10.1 - 12.6
Sepsis	245	5.2	4.6 - 5.8	150	6.0	5.0 - 6.9
COPD	2,428	51.5	50.1 - 52.9	1,387	55.1	53.1 - 57.0
GERD	1,321	28.0	26.7 - 29.3	761	30.2	28.4 - 32.0
Type 2 Diabetes Mellitus	1,533	32.5	31.2 - 33.8	846	33.6	31.8 - 35.4
Obstructive Sleep Apnea	383	8.1	7.3 - 8.9	224	8.9	7.8 - 10.0
Bronchitis	1,911	40.5	39.1 - 41.9	1,098	43.6	41.7 - 45.5
Upper Respiratory Tract Infection	451	9.6	8.7 - 10.4	266	10.6	9.4 - 11.8
Pulmonary Rehabilitation	76	1.6	1.3 - 2.0	52	2.1	1.5 - 2.6
Acute Coronary Syndrome	146	3.1	2.6 - 3.6	82	3.3	2.6 - 3.9
Angina Pectoris	256	5.4	4.8 - 6.1	153	6.1	5.1 - 7.0
Bleeding	567	12.0	11.1 - 13.0	319	12.7	11.4 - 14.0
Major Bleeding (Upper GI)	75	1.6	1.2 - 1.9	40	1.6	1.1 - 2.1
Major Bleeding (Lower GI)	312	6.6	5.9 - 7.3	180	7.1	6.1 - 8.2
Hemorrhage of the Rectum or Anus	118	2.5	2.1 - 2.9	69	2.7	2.1 - 3.4
Blood in Stool	127	2.7	2.2 - 3.2	62	2.5	1.9 - 3.1
Epistaxis	120	2.5	2.1 - 3.0	71	2.8	2.2 - 3.5
Hemorrhoids	106	2.2	1.8 - 2.7	57	2.3	1.7 - 2.8
Hemorrhoids with Bleeding	30	0.6	0.4 - 0.9	16	0.6	0.3 - 0.9
Intracranial Hemorrhage	39	0.8	0.6 - 1.1	19	0.8	0.4 - 1.1
Acute Pancreatitis	47	1.0	0.7 - 1.3	27	1.1	0.7 - 1.5
Hepatic Failure	14	0.3	0.1 - 0.5	7	0.3	0.1 - 0.5
Acute Renal Failure	615	13.0	12.1 - 14.0	378	15.0	13.6 - 16.4
Depression (MDD only)	575	12.2	11.3 - 13.1	315	12.5	11.2 - 13.8
Depression (MDD and other)	648	13.7	12.8 - 14.7	355	14.1	12.7 - 15.5

Optum Research Database – Medicare Advantage Population. Cohort Entry: 01 January 2008 - 30 September 2014

Abbreviations: *IPF* idiopathic pulmonary fibrosis, *CI* confidence Interval, *GI* gastrointestinal, *COPD* chronic obstructive pulmonary disease, *GERD* gastroesophageal reflux disease, *MDD* major depressive disorder

^a^Patients with 2+ claims with ICD-9 code of 410.x0 or 410.x1, primary position only. Patients with only one claim with these AMI codes but have claims suggestive of death were classified as AMI cases. Claims must be within 7 days of each other and must be from the inpatient or emergency room setting

^b^Patients with an ICD-9-CM code for AMI (410.x0, 410.x1) in the principal (or primary) diagnosis position on at least one facility claim for hospitalization. Claims from emergency departments were not be included in the case identification as they are likely to lead to misclassification

^c^ICD-9 code 430, 431, 433.x1, or 434.x1, only in the primary or secondary diagnosis position. For patients identified by 430, 431, exclude if the following ICD-9 diagnosis codes are present on the same day: 800.xx-804.xx, 850.xx-854.xx (in any position); or V57.xx (in the primary position)

^d^ICD-9 code 430, 431, 433.x1 or 434.x1, restricted to the primary diagnosis position on at least 1 facility claim for hospitalization

The incidence of the outcomes for the IPF cohort and IPF diagnostic testing subgroup are summarized in Table 5. In the IPF cohort, the IRs of the primary outcomes during follow-up ranged from 1.0/1,000 pys for lung transplantation to 180.4/1,000 pys for all-cause mortality. IRs of the secondary outcomes ranged from 3.7/1,000 pys for hepatic failure to 374.3/1,000 pys for arterial hypertension, respectively. Overall, IRs of most of the primary and secondary outcomes were slightly lower in the IPF cohort relative to the IPF diagnostic testing subgroup.

**Table 5:** Frequency and incidence of outcomes^a,b^ among the IPF cohorts during follow-up

	Restricted to Patients without the Condition during Baseline									
	IPF Cohort (*N*= 4,716)					IPF Diagnostic Testing Subgroup (*N*= 2,518)				
	Total Patients Eligible for Outcome	# of Patients with Outcome	Person-Years	IR	95% CI	Total Patients Eligible for Outcome	# of Patients with Outcome	Person-Years	IR	95% CI
Primary Outcomes										
Acute Respiratory Worsening of Unknown Cause	4,590	110	5,803	19.0	15.6 - 22.8	2,392	61	2,915	20.9	16.0 - 26.9
Pulmonary Hypertension	4,497	260	5,654	46.0	40.6 - 51.9	2,357	145	2,862	50.7	42.8 - 59.6
Pulmonary Arterial Hypertension	4,706	13	5,976	2.2	1.2 - 3.7	2,513	6	3,062	2.0	0.7 - 4.3
Lung Transplantation^c^	4,706	6	5,980	1.0	0.4 - 2.2	2,516	3	3,074	1.0	0.2 - 2.9
Lung Cancer	3,959	130	5,003	26.0	21.7 - 30.9	2,025	80	2,431	32.9	26.1 - 41.0
Acute Myocardial Infarction^d^	4,591	199	5,784	34.4	29.8 - 39.5	2,440	108	2,950	36.6	30.0 - 44.2
Acute Myocardial Infarction^e^	4,588	156	5,779	27.0	22.9 - 31.6	2,446	88	2,956	29.8	23.9 - 36.7
All-Cause Mortality	4,716	1,082	5,998	180.4	169.8 - 191.5	2,518	584	3,076	189.9	174.8 - 205.9
Secondary Outcomes										
GI Perforation	4,705	30	5,952	5.0	3.4 - 7.2	2,512	15	3,058	4.9	2.7 - 8.1
Chronic Renal Failure/Insufficiency	3,466	633	4,141	152.9	141.2 - 165.2	1,800	315	2,092	150.6	134.4 - 168.2
Hemorrhagic Diathesis or Coagulopathy	4,596	135	5,763	23.4	19.6 - 27.7	2,438	73	2,939	24.8	19.5 - 31.2
Venous Thrombosis	4,412	259	5,454	47.5	41.9 - 53.6	2,328	151	2,755	54.8	46.4 - 64.3
Pulmonary Embolism	4,561	143	5,712	25.0	21.1 - 29.5	2,420	77	2,916	26.4	20.8 - 33.0
Stroke^f^	4,537	187	5,631	33.2	28.6 - 38.3	2,427	96	2,895	33.2	26.9 - 40.5
Stroke^g^	4,637	129	5,778	22.3	18.6 - 26.5	2,479	64	2,963	21.6	16.6 - 27.6
Cardiac Arrhythmia	3,104	640	3,594	178.1	164.5 - 192.4	1,583	315	1,772	177.8	158.7 - 198.6
Congestive Heart Failure	3,221	641	3,948	162.4	150.0 - 175.4	1,669	322	1,981	162.5	145.3 - 181.3
Ischemic Heart Disease	2,810	494	3,197	154.5	141.2 - 168.8	1,445	249	1,569	158.7	139.6 - 179.7
Arterial Hypertension	1,117	401	1,071	374.3	338.6 - 412.8	577	197	516	382.0	330.5 - 439.2
Neutropenia	4,676	55	5,920	9.3	7.0 - 12.1	2,486	29	3,021	9.6	6.4 - 13.8
Pneumonia	4,280	382	5,294	72.2	65.1 - 79.8	2,232	189	2,670	70.8	61.0 - 81.6
Sepsis	4,471	348	5,610	62.0	55.7 - 68.9	2,368	166	2,873	57.8	49.3 - 67.3
COPD	2,288	607	2,457	247.1	227.8 - 267.5	1,131	298	1,150	259.1	230.5 - 290.2
GERD	3,395	565	3,657	154.5	142.0 - 167.8	1,757	289	1,814	159.3	141.5 - 178.8
Type 2 Diabetes Mellitus	3,183	234	3,947	59.3	51.9 - 67.4	1,672	121	2,003	60.4	50.1 - 72.2
Obstructive sleep apnea	4,333	176	5,329	33.0	28.3 - 38.3	2,294	94	2,703	34.8	28.1 - 42.6
Bronchitis	2,805	697	2,857	243.9	226.2 - 262.7	1,420	340	1,365	249.1	223.3 - 277.0
Upper Respiratory Tract Infection	4,265	337	4,960	67.9	60.9 - 75.6	2,252	181	2,504	72.3	62.1 - 83.6
Pulmonary Rehabilitation	4,640	130	5,773	22.5	18.8 - 26.7	2,466	85	2,934	29.0	23.1 - 35.8
Acute Coronary Syndrome	4,570	138	5,647	24.4	20.5 - 28.9	2,436	73	2,894	25.2	19.8 - 31.7
Angina Pectoris	4,460	151	5,493	27.5	23.3 - 32.2	2,365	81	2,795	29.0	23.0 - 36.0
Bleeding	4,149	550	4,758	115.6	106.1 - 125.7	2,199	274	2,434	112.6	99.7 - 126.7
Major GI Bleeding (Upper)	4,641	89	5,842	15.2	12.2 - 18.7	2,478	47	2,994	15.7	11.5 - 20.9
Major GI Bleeding (Lower)	4,404	311	5,325	58.4	52.1 - 65.3	2,338	165	2,727	60.5	51.6 - 70.5
Hemorrhage of the Rectum or Anus	4,598	129	5,708	22.6	18.9 - 26.9	2,449	65	2,915	22.3	17.2 - 28.4
Blood in Stool	4,589	164	5,663	29.0	24.7 - 33.7	2,456	85	2,920	29.1	23.3 - 36.0
Epistaxis	4,596	144	5,708	25.2	21.3 - 29.7	2,447	70	2,925	23.9	18.7 - 30.2
Hemorrhoids	4,610	114	5,725	19.9	16.4 - 23.9	2,461	57	2,941	19.4	14.7 - 25.1
Hemorrhoidal Bleeding	4,686	31	5,920	5.2	3.6 - 7.4	2,502	16	3,038	5.3	3.0 - 8.6
Intracranial Hemorrhage	4,677	58	5,913	9.8	7.4 - 12.7	2,499	35	3,021	11.6	8.1 - 16.1
Acute Pancreatitis	4,669	37	5,910	6.3	4.4 - 8.6	2,491	14	3,041	4.6	2.5 - 7.7
Hepatic Failure	4,702	22	5,990	3.7	2.3 - 5.6	2,511	11	3,072	3.6	1.8 - 6.4
Acute Renal Failure	4,101	506	5,189	97.5	89.2 - 106.4	2,140	261	2,630	99.2	87.6 - 112.0
Depression (MDD only)	4,141	398	4,964	80.2	72.5 - 88.5	2,203	208	2,531	82.2	71.4 - 94.1
Depression (MDD and other)	4,068	420	4,831	86.9	78.8 - 95.7	2,163	229	2,456	93.3	81.6 - 106.1

Optum Research Database – Medicare Advantage Population

Cohort Entry: 01 January 2008 - 30 September 2014

Abbreviations: *IR* incidence rate per 1,000 person-years, *CI* confidence Interval, *IPF* idiopathic pulmonary fibrosis, *COPD* chronic obstructive pulmonary disease, *GERD*, gastroesophageal reflux disease, *MDD* major depressive disorder;

^a^The mean follow-up time was 0.8 years (IPF cohort) and 0.7 years subgroup)

^b^Occurrence of one outcome did not preclude the occurrence of another, with the exception of all-cause mortality, which would result in censoring all additional follow-up

^c^For lung transplantation, patients with unilateral lung transplantation during the baseline period could be "at-risk" to receive another unilateral lung transplantation during the follow-up period; however, double lung transplantations during the baseline would preclude subsequent "at-risk" person-time during the follow-up period

^d^Patients with 2+ claims with ICD-9 code of 410.x0 or 410.x1, primary position only. Patients with only one claim with these AMI codes but have claims suggestive of death were classified as AMI cases. Claims must be within 7 days of each other and must be from the inpatient or emergency room setting

^e^Patients with an ICD-9-CM code for AMI (410.x0, 410.x1) in the principal (or primary) diagnosis position on at least one facility claim for hospitalization. Claims from emergency departments were not be included in the case identification as they are likely to lead to misclassification

^f^ICD-9 code 430, 431, 433.x1, or 434.x1, only in the primary or secondary diagnosis position. For patients identified by 430, 431, exclude if the following ICD-9 diagnosis codes are present on the same day: 800.xx-804.xx, 850.xx-854.xx (in any position); or V57.xx (in the primary position)

^g^ICD-9 code 430, 431, 433.x1 or 434.x1, restricted to the primary diagnosis position on at least 1 facility claim for hospitalization

The results were not substantially affected when patients who had claims during follow-up for other known causes of ILD (*i.e.,* claims suggesting they were misdiagnosed as IPF at cohort entry) were excluded (n=808, 17%, data not shown). One notable exception was that this subset of patients had a higher all-cause mortality rate (201.7/1,000 pys).

## Discussion

The results of this study suggest that IPF patients aged 65 years and above have a high morbidity and mortality. It included a broad range of comorbidities and outcomes, some of which have only been rarely or not been yet characterized in IPF populations. In particular, no published studies were found that included the prevalence or incidence of outcomes such as hepatic failure, acute pancreatitis or acute renal failure.

The patients included in this study are from a wide range of providers covered by Medicare, not restricted to major medical facilities, thus also reflecting diagnoses given by providers in general practice. Within the incident IPF cohort of 4,716 patients, over one half of the patients had a procedure for IPF diagnostic testing during baseline, the vast majority of which was HRCT rather than surgical biopsy. Surgical lung biopsy is an invasive diagnostic test; both HRCT and lung biopsy would tend to be performed only in larger medical settings. Although surgical lung biopsy was recommended by the 2011 international guidelines for the confirmation of the IPF diagnosis in patients who have a possible or probable UIP pattern on HRCT [3], not all patients are eligible or willing to undergo that procedure. As this study includes centers that are not necessarily ILD referral centers, there is the potential that these centers are not so experienced and comfortable to perform this invasive procedure. The subgroup analysis only looks at the diagnostic procedures done at baseline (i.e. until the first medical claim of IPF). In the majority of cases the surgical lung biopsy is only done after the result of the HRCT (in cases of possible or probable UIP) – which means the surgical lung biopsy would only be done after the baseline period and therefore not captured in the definition of the cohort/subgroup. Unlike clinical trials [5] that include a higher proportion of males, the gender distribution in this study is consistent with other publications on IPF populations, where approximately 50% of the patients were female [26, 27]. This may reflect women’s greater likelihood to seek health-care services, therefore are more frequently observed in insurance claim databases [26].

Changes in the ICD-9 codes for IPF in October of 2011 did not influence the baseline characteristics of patients identified. However, if coding practice changes were implemented gradually, then some possible IPF patients with claims on or after October 2011 who were coded with the pre-October 2011 code (516.3) would have been excluded.

Comparisons to other publications should consider that study findings are influenced by the complexity of the IPF cohort definition, different distributions of age and sex, the coding used to define comorbidities, length of follow-up and the different underlying databases. Nevertheless, the reported prevalence and incidence rates observed in this study fell within the range of estimates reported in other publications. A systematic literature review [28] of the prevalence of pulmonary and extra pulmonary comorbidities among IPF patients included several of the outcomes included in this study and estimates ranged from 3-86% for PH, 3-48% for lung cancer, 6-91% for sleep apnea (various types), 6-67% for COPD, 6-68% for ischemic heart disease (IHD) and 0-94% for GERD. Our reported prevalence estimates fell within these ranges, with baseline prevalence of 4.6% for PH, 16.1% for lung cancer, 8.1% for obstructive sleep apnea, 51.5% for COPD, 40.4% for IHD and 28.0% for GERD.

Of particular interest for IPF populations is the occurrence of exacerbations of IPF. The percent of patients in this cohort with ARWUC during follow-up was rather low with 2.4% (95% CI: 2.0-2.8, data not shown). In comparison, a retrospective study of data collected from 461 patients with diagnosed IPF reported an annual percent of 14.2% of clinically defined acute respiratory worsening [29]. Reports in clinical trials have tended to be lower than this, and Raghu et al reported that ARW are believed to occur in between 5 and 10% of IPF patients per year [29]. The incidence of ARWUC is difficult to establish in claims data due to variations in methodologies used in different studies [29] and the numerous exclusionary comorbidities included in the definition, such as left heart failure, pulmonary embolism and other identifiable causes of lung injury. In addition, dyspnea, an essential component of the clinical definition of ARW may not be well-captured in claims data, leading to underestimation of the condition. This ARWUC algorithm is a proxy for clinically defined ARW and further validation is desirable.

Relative to findings reported in a similar study based on Optum’s commercially insured population [30], the IPF cohort in this study population has substantially higher morbidity and mortality. This is likely due to the fact that the study population of this study was restricted to Medicare-eligible patients aged 65 years and above, while the previous study included IPF patients aged 40 years and older. Primary outcomes with notably higher IRs (per 1,000 pys) included AMI (34.4 vs 13.8), pulmonary hypertension (46.0 vs. 22.5), lung cancer (26.0 vs. 17.6), and mortality (180.4 vs. 97.1). The IR for lung transplantation was lower (1.0 vs. 6.0), likely due to the practice in the US during this study period of restricting lung transplantation to patients under the age of 65 years [31]. IRs of most of the secondary outcomes were higher among the elderly population, in particular for chronic renal failure, congestive heart failure, pulmonary rehabilitation and intracranial hemorrhage, all of which were more than twice as high. In both studies, arterial hypertension was the secondary outcome with the highest IR and the most prevalent condition during baseline (76.3% in elderly, (Table 5), 55.3% in commercial (data not shown)). Although arterial hypertension is known to be highly prevalent in the US population, the high IR may also be due to the broad range of codes used to define this outcome [16, 17].

Collard et al [27] performed a similar analysis using US claims data from commercially insured and Medicare patients. The Collard et al [27] population was not restricted to the Medicare population (20.8% were 64 years or younger) and many of the outcome definitions were less inclusive and consequently, IRs (per 1,000 pys) were consistently lower than those reported in the Optum population: heart failure (67.5 vs. 162.4), bronchitis (31.5 vs. 243.9), AMI (21.7 vs. 34.4), diabetes (18.0 vs. 59.3), lung cancer (11.9 vs. 26.0), pulmonary embolism (10.7 vs. 25.0), GERD (7.5 vs. 154.5), pulmonary hypertension (6.8 vs. 46.0), depression (6.3 vs. 80.2 for MMD only) and sleep apnea (1.2 vs. 33.0).

Raimundo et al [32] also used insurance claims data to examine the impact of IPF on HCRU, separately in 2009, 2010 and 2011.The range of mean age of the cohorts was 69.8-71.3 and approximately 50% of each cohort was male. In comparison, our study population had higher proportion of patients hospitalized in the past year (51% vs. 37%), lower number of outpatient visits (14.8 vs. 18.5), lower use of oral corticosteroids (33.4% vs. 44.6%).

This study has some key limitations. The study was done using automated medical and prescription claims. While claims data are extremely valuable for the efficient examination of health care outcomes and utilization, all claims databases have certain inherent limitations. Presence of a diagnosis code on a medical claim is not necessarily positive presence of disease, as it may be incorrectly coded or included as rule-out criteria rather than actual disease. Similarly, claims for diagnostic tests may be observed but results are not reported in claims data. We restricted the eligibility criteria to having a one-year look back for identification of incident cases of IPF, so patients who had an earlier diagnosis of IPF but not in the one-year lookback could have been misclassified as incident. However, this is unlikely as IPF is a chronic condition and it is highly likely that patients would be seeking health care during that one-year look back period. These limitations may lead to potential misclassification and impact this study due to the difficulty in diagnosing IPF when patients first present to medical providers. Sensitivity and subgroup analyses were implemented to evaluate the impact that different selection criteria had on cohort characteristics, specifically creating a stricter cohort definition for the subgroup analysis which includes only those patients who had IPF diagnostic testing in line with the recommendations of the international diagnostic guidelines [3].

True incidence is difficult to identify, as it cannot be determined whether the code pertains to newly observed conditions or for care related to the conditions that were observed at an earlier (and possibly unobserved) date. Although this study focuses on the IR among patients without the condition observed in baseline, this may still result in the overestimation of rates of outcomes such as chronic and acute renal failure, which are captured by a broad range of non-specific codes, resulting in some of the highest incidence rates observed in this study. Some outcomes, such as epistaxis or other bleeding events may not require medical attention and therefore are not well captured in medical claims, leading to underestimation of those events. Medications that can be obtained without prescriptions (*i.e.,* over-the-counter medications) are not observed in claims data. Medications that are given during an inpatient stay are also not captured, leading to underestimation of their use. In addition, given that there were no efficacious or approved treatments available during this study period other than lung transplantation, patients may have received additional (but unobserved in claims data) health care and medications through involvement in clinical trials.

Duration of follow-up can be limited in the Optum-MAPD database due to individuals changing into more traditional Medicare coverages or Medicare Advantage plans administered by other insurance companies. Thus, outcomes that occur after enrollment ends would not contribute to the prevalence or incidence rates. Although not systematically evaluated, there is a suggestion that as patients get sicker, they are more likely to disenroll from Medicare Advantage plans [33, 34].

Historically, the SSA DMF provided about 90% coverage for patients older than 65 years. Starting on 01 November 2011, the SSA determined that protected state records could no longer be disclosed. Section 205(r) of the Social Security Act prohibited the SSA from disclosing state death records received through their contracts with these states, except in limited circumstances. This has resulted in the reduction of available records on the public DMF by 1 million annually (about 30% reduction) [35]. Some deaths may not be available in the DMF, and out-of-hospital deaths may be not be identified, potentially leading to underestimation of the mortality incidence rate.

There are several advantages to conducting this study in the Optum-MAPD. Unlike site-based or registry-based studies that are typically limited in population sample size, the Optum-MAPD contains millions of lives, allowing for broader investigations of drug use patterns and disease outcomes. This is especially valuable for investigating rare outcomes in a population with a rare disease such as IPF. Underlying information is geographically diverse across the US. Relative to the overall US Medicare population, the Optum MAPD population has a similar distribution of gender and age, members from the Northeast and Midwest, and proportion of members who are African-American, Hispanic or Asian. It has a higher proportion of members from the South and fewer from the West and a higher proportion with race categorized as other/unknown. The average length of enrollment in the MAPD is almost 5 years for this age group.

## Conclusions

Elderly IPF patients experience a variety of comorbidities, both before and after diagnosis. This study helps to gain a better understanding of the outcomes of this disease, which is important to optimize management of this patient population and thus improve disease outcomes. The cohort characteristics were not affected by modifications to the cohort definition, suggesting that the definition of IPF captures similar patients despite variations in the operational definition of IPF. Therapies for IPF and for the associated comorbidities may reduce morbidity and associated HCRU of these patients.

## Additional file

Additional file 1: ICD-9-CM Codes For Interstitial Lung Diseases Other Than Idiopathic Pulmonary Fibrosis (DOCX 18 kb)

## Abbreviations

AMI: Acute myocardial infarction
ARW: Acute respiratory worsening
ARWUC: Acute respiratory worsening of unknown cause
CI: Confidence interval
COPD: Chronic obstructive pulmonary disease
DMF: Death master file
EBV: Epstein-Barr virus
FDA: Food and Drug Administration
GERD: Gastroesophageal reflux disease
GI: Gastrointestinal
HCRU: Health care utilization
HRCT: High-resolution computed tomography
IHD: Ischemic heart disease
ILD: Interstitial lung disease
IPF: Idiopathic pulmonary fibrosis
IQR: Interquartile range
IR: Incidence rate
MAPD: Medicare Advantage and Part D plan
MDD: Major depressive disorder
NAC: N-acetyl cysteine
PAH: Pulmonary arterial hypertension
PH: Pulmonary hypertension
pys: Person-years
SD: Standard deviation
SLB: Surgical lung biopsy
SSA: Social Security Administration
UIP: Interstitial pneumonia
US: United States
