# Supplementary material for: Characterizing idiopathic pulmonary fibrosis patients using US Medicare-advantage health plan claims data
Source: BMC Pulm Med. 2019 Jan 10;19:11. doi: 10.1186/s12890-018-0759-5 (PMC6327584; doi:10.1186/s12890-018-0759-5)
Supplement: Supplementary file 1 — ICD-9-CM Codes For Interstitial Lung Diseases Other Than Idiopathic Pulmonary Fibrosis (DOCX 18 kb) [file 12890_2018_759_MOESM1_ESM.docx]

| **Additional File 1. ICD-9-CM Codes For Interstitial Lung Diseases Other Than Idiopathic Pulmonary Fibrosis** | | | |
| --- | --- | --- | --- |
| 135 | Sarcoidosis |  |  |
| 237.7 | Neurofibromatosis |  |  |
| 237.70 | Neurofibromatosis, unspecified | | |
| 237.71 | Neurofibromatosis, Type 1 | |  |
| 237.72 | Neurofibromatosis, Type 2 | |  |
| 237.73 | Schwannomatosis |  |  |
| 237.79 | Other Neurofibromatosis | |  |
| 272.7 | Lipidoses |  |  |
| 277.3 | Amyloidosis |  |  |
| 277.30 | Amyloidosis, unspecified | |  |
| 277.31 | Familial Mediterranean fever | |  |
| 277.39 | Other amyloidosis |  |  |
| 277.8 | Other specified disorders of metabolism-includes eosinophilic granuloma | | |
| 277.80 | Other specified disorders of metabolism | | |
| 277.81 | Primary carnitine deficiency | |  |
| 277.82 | Carnitine deficiency due to inborn errors of metabolism | | |
| 277.83 | Iatrogenic carnitine deficiency | | |
| 277.84 | Other secondary carnitine deficiency | | |
| 277.85 | Disorders of fatty acid oxidation | | |
| 277.86 | Paroxisomal disorders | |  |
| 277.87 | Disorders of mitochondrial metabolism | | |
| 277.88 | Tumor lysis syndrome | |  |
| 446.21 | Goodpasture's syndrome | |  |
| 446.4 | Wegener's granulomatosis | |  |
| 495 | Extrinsic allergic alveolitis | |  |
| 495.0 | Farmers' lung |  |  |
| 495.1 | Bagassosis |  |  |
| 495.2 | Bird Fanciers' lung |  |  |
| 495.3 | Suberosis |  |  |
| 495.4 | Malt workers' lung |  |  |
| 495.5 | Mushroom workers' lung | |  |
| 495.6 | Maple bark-strippers' lung | |  |
| 495.7 | Ventilation pneumonia | |  |
| 495.8 | Other specified allergic alveolotis | | |
| 500 | Coal workers' pneumoconiosis | | |
| 501 | Asbestosis |  |  |
| 502 | Pneumoconiosis due to other silica or silicates | | |
| 503 | Pneumoconiosis due to other inorganic dust | | |
| 504 | Pneumoconiosis due to inhalation of other dust | | |
| 505 | Pneumoconiosis, unspecified | |  |
| 506.4 | Chronic respiratory conditions due to fumes or vapors | | |
| 508.1 | Chronic and other pulmonary manifestations due to radiation | | |
| 508.8 | Respiratory conditions due to other specified external agents | | |
| 516 | Pulmonary alveolar proteinosis | | |
| 516.0 | Pulmonary alveolar hemosiderosis | | |
| 516.1 | ldiopathic pulmonary hemosiderosis | | |
| 516.2 | Pulmonary alveolar microlithiasis | | |
| 516.4 | Lymphangioleiomyomatosis | |  |
| 516.8 | Other specified alveolar and parietoalveolar pneumonopathies | | |
| 516.9 | Unspecified alveolar and parietoalveolar pneumonopathies | | |
| 517.2 | Lung involvement in systemic sclerosis | | |
| 517.8 | Lung involvement in other diseases classified elsewhere | | |
| 518.3 | Pulmonary eosinophilia | |  |
| 555 | Regional enteritis |  |  |
| 555.0 | Regional enteritis, Small intestine | | |
| 555.1 | Regional enteritis, Large intestine | | |
| 555.2 | Regional enteritis, Small intestine with large intestine | | |
| 710 | Diffuse diseases of connective tissue | | |
| 710.0 | Systemic lupus erythematosus | | |
| 710.1 | Systemic sclerosis |  |  |
| 710.2 | Sjӧgren's disease |  |  |
| 710.3 | Dermatomyositis |  |  |
| 710.4 | Polymyositis |  |  |
| 714 | Rheumatoid Arthritis and other inflammatory polyarthropathies | | |
| 714.0 | Rheumatoid Arthritis | |  |
| 714.1 | Felty's syndrome |  |  |
| 714.2 | Other rheumatoid arthritis with visceral or systemic involvement | | |
| 714.3 | Juvenile chronic polyarthritis | |  |
| 714.30 | Polyarticular juvenile rheumatoid arthritis, chronic or unspecified | | |
| 714.31 | Polyarticular juvenile rheumatoid arthritis | | |
| 714.32 | Pauciarticular juvenile rheumatoid arthritis | | |
| 714.33 | Monoarticular juvenile rheumatoid arthritis | | |
| 714.4 | Chronic postrheumatic arthropathy | | |
| 714.8 | Other specified inflammatory polyarthropathies | | |
| 714.81 | Rheumatoid lung |  |  |
| 714.89 | Other specified inflammatory polyarthropathies, other | | |
| 714.9 | Unspecified inflammatory polyarthropy | | |
| 720 | Ankylosing spondylitis and other inflammatory spondylopathies | | |
| 720.0 | Ankylosing spondylitis | |  |
| 720.1 | Spinal enthesopathy | |  |
| 720.2 | Sacroliitis, not elsewhere classified | | |
| 720.8 | Other inflammatory spondylopathies | | |
| 720.81 | Inflammatory spondylopathies in diseases classified elsewhere | | |
| 720.89 | Inflammatory spondylopathies, other | | |
| 720.9 | Unspecified inflammatory spondylopathy | | |
| 759.5 | Tuberous sclerosis |  |  |
| For time periods after October 2011 when the ICD-9 codes changed for IPF, these codes were added to the list of ILDs other than IPF. | | | |
| 516.3 | Idiopathic interstitial pneumonia | | |
| 516.30 | Idiopathic interstitial pneumonia, not otherwise specified | | |
| 516.32 | Idiopathic non-specific interstitial pneumonitis | | |
| 516.33 | Acute interstitial pneumonitis | |  |
| 516.34 | Respiratory bronchiolitis interstitial lung disease | | |
| 516.35 | Idiopathic lymphoid interstitial pneumonia | | |
| 516.36 | Cryptogenic organizing pneumonia | | |
| 516.37 | Desquamative interstitial pneumonia | | |

.
